# Visualizing defects and pore connectivity within metal–organic frameworks by X-ray transmission tomography†

**DOI:** 10.1039/d1sc00607j

**Published:** 2021-05-06

**Authors:** Rafael Mayorga-González, Miguel Rivera-Torrente, Nikolaos Nikolopoulos, Koen W. Bossers, Roozbeh Valadian, Joaquín Yus, Beatriz Seoane, Bert M. Weckhuysen, Florian Meirer

**Affiliations:** Inorganic Chemistry and Catalysis Group, Debye Institute for Nanomaterials Science, Utrecht University Universiteitsweg 99 3584 CG Utrecht The Netherlands b.m.weckhuysen@uu.nl f.meirer@uu.nl; Instituto de Cerámica y Vidrio, Consejo Superior de Investigaciones Científicas (CSIC) Kelsen 5 28049 Madrid Spain

## Abstract

Metal–Organic Frameworks (MOFs) have the potential to change the landscape of molecular separations in chemical processes owing to their ability of selectively binding molecules. Their molecular sorting properties generally rely on the micro- and meso-pore structure, as well as on the presence of coordinatively unsaturated sites that interact with the different chemical species present in the feed. In this work, we show a first-of-its-kind tomographic imaging of the crystal morphology of a metal–organic framework by means of transmission X-ray microscopy (TXM), including a detailed data reconstruction and processing approach. Corroboration with Focused Ion Beam-Scanning Electron Microscopy (FIB-SEM) images shows the potential of this strategy for further (non-destructively) assessing the inner architecture of MOF crystals. By doing this, we have unraveled the presence of large voids in the internal structure of a MIL-47(V) crystal, which are typically thought of as rather homogeneous lattices. This challenges the established opinion that hydrothermal syntheses yield relatively defect-free material and sheds further light on the internal morphology of crystals.

## Abstract 2

TXM-tomography unraveled large macropore defects within a MIL-47(V) MOF crystal. These pores do not seem to be well connected and they show a preferential orientation.

## Graphical abstract

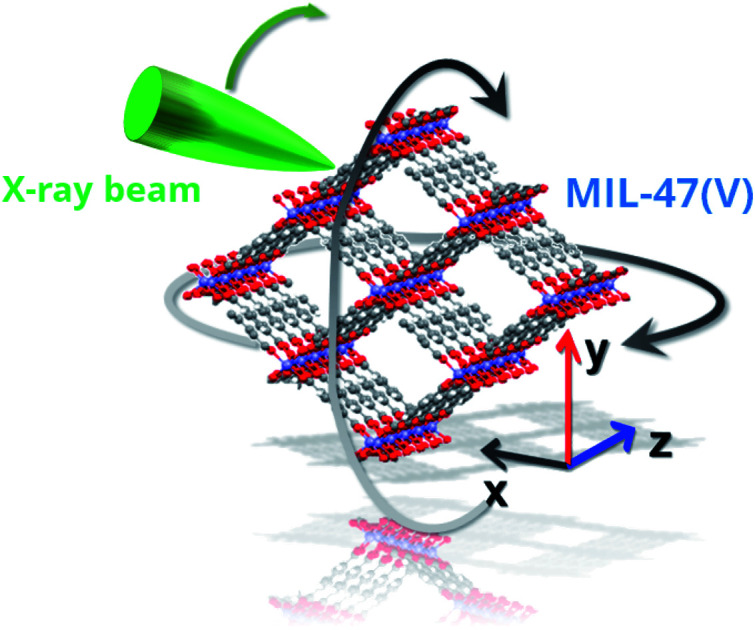

## Introduction

Metal–Organic Frameworks (MOFs) have become a very important group of multifunctional porous materials, showing the first commercial applications after years of intense fundamental and more applied research.^1–4^ A broad array of uses, ranging from biomedicine and drug delivery to chemical sensing or catalysis have been extensively studied.^5–8^ Perhaps two of the most studied applications are adsorption and purification of gaseous and liquid streams, such as hydrocarbon, *e.g.* olefin/paraffin, mixtures in refinery processes, residual traces, *i.e.* CO_*x*_, NO_*x*_ or SO_*x*_, or the removal of contaminants from water.^5,9–12^ The high performance in such applications arises from their extremely high surface areas (often >1000 m^2^ g^−1^), regular pore systems that allow for (in principle) homogeneous diffusion, and the presence of interacting metal sites that may undergo reversible redox processes.^13–17^ This combination renders MOFs as materials of choice for advanced separations,^18^ being particularly interesting for mixed-matrix membranes.^19–21^

More specifically, many studies concerning diffusion and surface interactions of CO_2_,^22–25^ H_2_,^26,27^ alkanes,^28–31^ alkenes,^32^ aromatic hydrocarbons^33–38^ within the pores of MIL-47(V) have been previously published. These works highlight that separation and sorption properties of MOFs heavily rely on weak and strong interactions between the adsorbates and the internal micropore surfaces, *i.e.* organic ligands and metal sites, of the crystal lattice. Thus, the formation of defects or hollow cavities at the meso- (*i.e.* pore sizes > 50 nm),^39^ which may happen along with the deposition of nanosized deposits of impurities (*e.g.* metallic or metal oxides) disrupting the 1D channels of the structure may lead to alternative diffusion pathways, thus altering the separation properties.^39^ Therefore, understanding of the meso- and macropore structure in MOFs is crucial for developing refined synthetic methods.

Examples for imaging pores in MOFs are still scarce and rely mainly on electron microscopy, which often implies beam damage,^40^ and are restricted, to the best of our knowledge, to a high-resolution transmission electron microscopy (HR-TEM) study of defect mesopores in UiO-66 crystals.^41^ Moreover, this technique remains restricted to volumes in the range of nm^3^, which prevents the user from probing large structures, *e.g.* a whole single-crystal. Other than that, fluorescence-lifetime imaging (FLIM) of UiO-66 tagged crystals has been recently reported, although rather than studying mesopore structure, the subject of matter was chemical heterogeneities.^42^ Several studies from multiple groups have shown how X-ray 3D imaging can be used for studying the porosity of porous materials at different length scales, overcoming the effects of severe beam damage and expanding the imaged volumes to the micron-range maintaining nanometer resolutions and allowing for tomography-based mass transfer simulations^43–49^ For instance, imaging of poisoning metal deposits within the pores of fluid catalytic cracking (FCC) particles with full-field transmission X-ray microscopy (TXM)^43,50,51^ and micro X-ray fluorescence (μ-XRF)^43,52–54^ allowed Kalirai and others to evaluate how the pores can become clogged during operation.^55^ Such studies require a 3D assessment and mapping of both deposits and the entire macropore space of individual catalyst particles with sizes in the 100 micrometre regime, that is, a method that provides both sufficient spatial resolution and a large field of view.

**Fig. 1:**
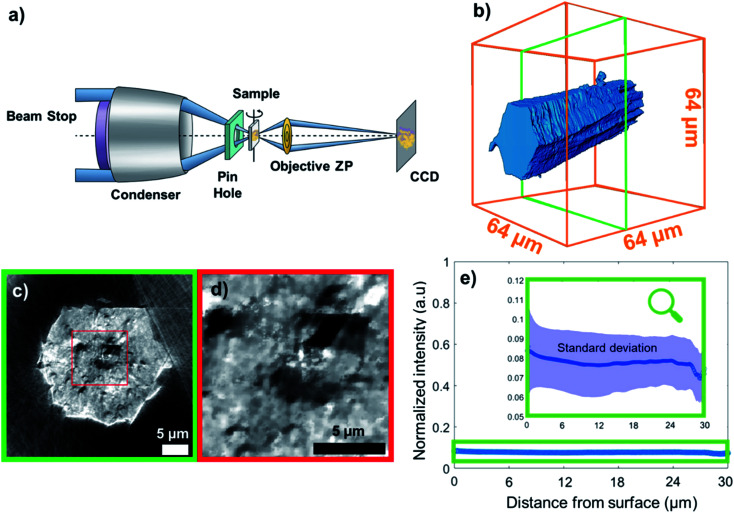
(a) Transmission X-ray microscope setup. ZP: zone plate, CCD: charge-coupled device detector. (b) 3D reconstruction of the metal organic framework (MOF) crystal under study, namely MIL-47(V), from X-ray transmission microscopy (TXM) data; (c) a cross section vertical to the crystal axis. If the chemical composition (*i.e.* ratio of elements) varies only slightly over the sample, then, the per voxel variation in measured X-ray absorption (*i.e.* the reconstructed voxel gray scale level) directly relates to variation in material density. Therefore, the darker regions of the cross section represent areas of much lower density (such as voids), whereas the more intense pixels correspond to dense areas. Note that we used the outer space of the capillary, *i.e.* no matter present, as the reference absorption value for the lower end. (d) Zoom-in to a region in (c). (e) Average grayscale intensity (min–max scaled) plotted as a function of distance from the particle surface; the shaded field corresponds to standard deviation. The graph reveals no correlation between average voxel intensity and distance from surface. However, the standard deviation of the intensity decreases with the distance from the surface indicating a higher density of defects closer to the surface (more details can be found in Section 14 of the ESI†).

Recently, a first X-ray 3D study of HKUST-1 MOFS was published by Ferreira-Sanchez *et al.*, addressing issues such as spatially-resolved metal speciation and by-product phase formation^56^ However, this work focused on chemical heterogeneities in the crystal and not on porosity. Despite the tremendous importance of this property in the applications in which MOFs are typically used, to the best of our knowledge, no detailed studies focusing on porosity have been reported yet. Herein, we made use of full-field transmission X-ray microscopy (TXM) nanotomography to image a single MIL-47(V) crystal in 3D and map the macropore defects present within (Fig. 1a). Moreover, pore network analysis allowed us to evaluate pore connectivity as well as preferential macro-pore orientation throughout the crystal. This allowed us to not only draw conclusions about the macro-pore architecture and its potential implications in diffusion-controlled operations, but also to formulate hypotheses on crystal growth and formation of the MIL-47(V) topology. These findings have been corroborated by Focused Ion Beam-Scanning Electron Microscopy (FIB-SEM) images on the same material.

## Results and discussion

### Tomographic reconstruction and macro-porosity

In this work, the terms porosity or pores always refer to macroporosity and macropores with dimensions above the estimated spatial resolution respectively in this article. Fig. 1b shows the 3D reconstruction of a MIL-47(V) needled-like crystal of ∼65 μm length. Fig. 1c and d, show a virtual slice of the reconstructed sample density corresponding to a cross-section, and a zoom-in of the voids, respectively. The latter shows areas of lower X-ray absorption intensity (darker), exhibiting macropores in the range from a few hundreds of nanometres to 2–3 μm in size. The upper end of this range is of great importance, because both bulk and intraparticle pores with such dimensions will be detected in Hg-intrusion measurements. The nature of such an overlap in pore size ranges for inter- and intraparticle porosity is unknown and it is impossible to discriminate between these two types of adsorption contributions in Hg-intrusion isotherms (Fig. S1†). This, in turn, prevents applying any corrections to the resulting overestimation in determined porosity. Beyond evidencing the presence of macropores, the X-ray absorption in each voxel is directly correlated with material density as the variation in element composition in this sample is negligible.

**Fig. 2:**
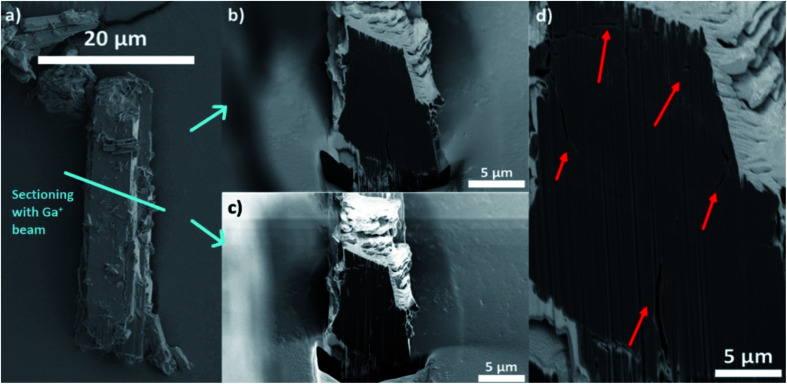
(a) SEM image of a MIL-47(V) MOF crystal synthesised in the same way as the one studied by TXM. (b) SEM image of FIB-cut cross section of the particle displayed in (a) showing macropores. (c) SEM image of the cross section shown in (b) imaged with secondary electrons accentuating the macropores (light regions). (d) Zoom-in of the micrograph displayed in (b). The red arrows indicate macropores observed in the cross-section of the crystal.

The data can therefore be used to qualitatively study differences in crystal defects even at resolutions smaller than the achieved spatial resolution of 230 nm. Fig. 1e shows the mean voxel intensity as a function of distance from the particle surface; here one can observe that on average the density of crystal defects changes only insignificantly as a function distance from the surface (correlation coefficient: 0.0990). However, the standard deviation (shaded area) of the intensity levels increases close to the surface hinting towards a higher density of defects (more details can be found in Section 14 of the ESI†). This higher intensity variance on the surface is due to the presence of less intense (porous) voxels as well as high intensity outlier regions (see Section 13 of the ESI†). Our hypothesis is that these high intensity regions correspond to vanadium clusters that precipitate during crystallization which would be in line with the findings of Ferreira-Sanchez *et al.*^56^ It is worth noting that a strong variation in the concentration of elements (atomic number, *Z*) could also cause such variation as the X-ray absorption coefficient (note that *μ* ∝ *Z*^4^), although the purity of the V precursor used was >99%. Hence, it seems unlikely the widespread presence of lighter or heavier elements throughout the crystal. To further validate these TXM-tomography results, six crystals were sectioned by using a focused Ga^+^ ion beam (FIB) and imaged by scanning electron microscopy (SEM). As shown in Fig. 2, also this method revealed macropores to be ubiquitously present. For FIB-SEM data the surface area of the observed macropores and the one of the cross-sections were segmented manually. Based on this, the porosity of the sample was estimated by calculating the fraction of pixels in the cross section identified as void space (see Fig. S2†). The estimated macroporosity of the different FIB cut cross sections varied strongly (Fig. 2, S2 and S3†). However, none of the observed cross sections displayed a porosity greater than 2%. While confirming findings from TXM, single FIB-SEM measurements can only provide 2D information, which prevents a detailed analysis of heterogeneity and pore connectivity. The latter is, however, crucial to understand how these macro-pores affect MOF performance in separation or adsorption.

### 3D image segmentation

**Fig. 3:**
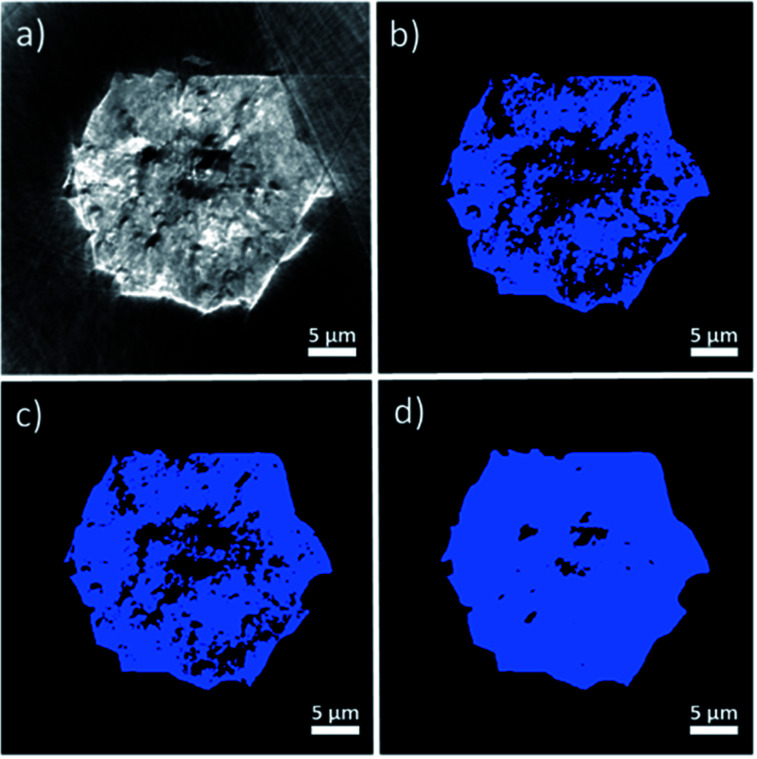
Virtual cross section through the MOF crystal as reconstructed from TXM tomography. (a) Grayscale image displaying the samples density distribution. (b) Binarization result based on a segmentation threshold based on Hg-porosimetry (29% total porosity). (c) Intensity threshold based on highest background intensity value (18% total porosity). (d) Intensity threshold based on mean of the background intensity values (2.6% total porosity).

In order to analyze the macropore network of the MOF crystal, it is necessary to classify each voxel of the reconstructed volume into either void or solid phase to create a binarized data volume. Therefore, a threshold value (*i.e.* a given grayscale intensity value) must be selected. Every voxel with a grayscale intensity above this threshold will then be considered as solid while voxels below this value will be assigned as void space. As this crucial parameter can in principle be chosen arbitrarily, additional knowledge for selecting a correct value is needed. The threshold value determines the void voxel fraction of the reconstructed particle volume and is therefore directly correlated to the total porosity determined from the reconstructed data. To have an initial estimate for total porosity and in turn the threshold value, Hg-porosimetry characterization of the same material was performed resulting in a total porosity of 29% (see section 1). However, visual inspection of the grayscale cross sections (Fig. 3a) and corresponding binarized images (Fig. 3b–d) suggests that this technique leads to an overestimation of porosity, as relatively few bright areas are classified as void space if this porosity value is applied.

The FIB-SEM cross sections shown in Fig. 2 and S3† exhibit much lower porosity values (<2%, after binarization and quantification of the two different types of pixels). This also suggests that the material porosity is much lower than the one estimated by Hg-intrusion. As discussed above, Hg-porosimetry cannot distinguish between inter- and intraparticle voids and interprets inter particle porosity as intra particle void space, leading to an overestimation of the actual void fraction. Therefore, in order to achieve a segmentation that better represents the actual macro-porosity of the crystal, a series of X-ray absorption thresholds was evaluated. As a first boundary case, the highest intensity value of the gray scale values determined in the region outside the crystal (*i.e.* X-ray absorption of the air surrounding the crystal) was used as a threshold value (see Fig. S4†). By doing this, about 18% of the particle volume was identified as void space.

However, also this value still seemed to overestimate macroporosity when the binarized cross sections (Fig. 3b–d) are compared to their grayscale counterparts (Fig. 3a). Besides, the FIB-SEM results suggest a much lower macroporosity than 18%. Finally, as a third, lower boundary case, an intensity threshold was chosen based on the average intensity of the image background (see Fig. S4†), which results in a total porosity of 2.6% (Fig. 3d). The porosity estimated with FIB-SEM (<2%) (Fig. S2†) is in a comparable region of this value. In addition, the binarized cross section image resulting from this threshold, seems to be in better alignment with the particle's X-ray absorption image (Fig. 3a). Therefore, we suggest that the total macro-porosity of the studied individual MOF crystal should be in the region of 2–3%.

Apart from the above-mentioned porosity overestimation, Hg-porosimetry has other limitations that lead to an underestimation of the pore volume. For example, closed pores cannot be probed as Hg cannot access them.^57^ In addition, this technique does not measure the pore size distribution, but the pore throat size distribution, that is the size of the smallest entrance of the accessible pores.^57^ For the sake of proving the accuracy of this measurement, a pore throat analysis as described in Section 5 of the ESI† was performed (assuming 29% porosity). This analysis was used to study the pore throat size distribution, shown in Fig. S6.† A single peak at around 200 nm is observed from the numerical computation, while the largest peaks in Hg-intrusion are visible at the interparticle space (2–3 μm) and 8–6 nm, ascribed to the mechanical compression phenomenon.^58^ This again supports the idea that Hg-porosimetry has certain pitfalls in studying the porosity of the material and highlights the advantage of being able to tune the threshold value by cross-validating the segmentation. In brief, by combining: (i) bulk Hg-porosimetry evaluating many particles but suffering from uncertainties based on interparticle porosity; (ii) FIB-SEM providing high spatial resolution but being limited to studying a few crystal cross-sections; and (iii) 3D TXM data of a single but whole individual MIL-47(V) MOF crystal; we obtained a reasonable estimate for the macro-porosity of the sample. To further exploit the advantages of TXM and building upon the ability to evaluate the effect of different porosity values on pore connectivity and crystal accessibility, we continued our pore-network analysis using not only one but a series of grayscale threshold values corresponding to macro-porosities of 29%, 25%, 20%, 18%, 15%, 10%, 5%, and 2.6%. In the following section, we highlighted the boundary cases of 29, 18, and 2.6% porosity.

### Connected macroporosity and intraparticle heterogeneity

**Fig. 4:**
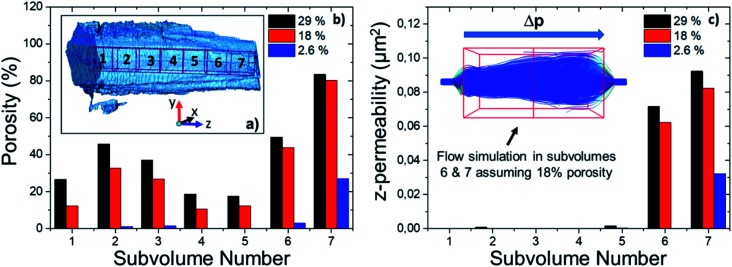
(a) Selected sub-regions (7.5 × 7.5 × 7.5 μm^3^) for porosity and permeability analysis. (b) Individual porosities of the 7 sub-volumes determined for different segmentation thresholds, *i.e.* total porosities of the whole crystal. (c) Permeabilities of subregions and simulated steady state flow streamlines in sub-volumes 6 and 7 assuming a total porosity of 18%, an incompressible fluid, and a negative pressure gradient from left to right.

**Fig. 5:**
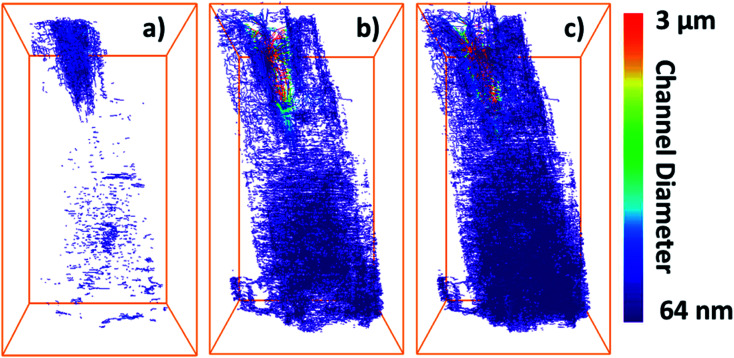
Determined pore network models of the MIL-47(V) crystal assuming total porosities of 2.6% (a) 18% (b) and 29% (c). The color scale represents the diameter of the pore channels: blue corresponds to small values, whereas red represents wider channels.

To assess the heterogeneity in macroporosity within the MOF crystal, the porosities of 7 sub-volumes of 7.5 × 7.5 × 7.5 μm^3^ along the primary MOF-crystal axis were computed (Fig. 4a). The flow permeability of a material can be used as a measure of its pore connectivity. If a porous material is not permeable, it consists only of isolated pores. By contrast, the more permeable the material the higher its pore connectivity. In order to evaluate how permeable was the reconstructed crystal, flow simulations were carried out on each of the sub-volumes using the Avizo® XLabHydro software (Fig. 4b). A more detailed explanation can be found in Section 6 of the ESI.† Then, the permeabilities and total porosities of all subvolumes were then compared. Remarkably, large intra-particle heterogeneity was revealed by this analysis: for example, when the threshold chosen for binarization was set to achieve a total porosity of 2.6%, some sub-regions show essentially no macro-porosity (1, 4, 5). However, sub-volume 7 has a void fraction of more than 11-fold the particle average (Fig. 4b). As the hypothetical total porosity was stepwise increased by setting higher thresholds, *i.e.* 5 10, 15, 18, 20, 25, and 29%, sub-volumes 1, 4, and 5 gradually exhibited increasing porosity (Fig. 4b). Yet, sub-volume 7 showed a porosity much higher than the total average for all the studied thresholds. Several sub-volumes exhibit porosity, but virtually no permeability (sub-regions 1–5). This is the case even when the total porosity of the MOF is assumed to be 29% (based on Hg-porosimetry). As discussed above, this porosity value strongly exceeds the real one. This means that the macropores within these regions are isolated even if their total porosities are artificially increased. On the other hand, sub-volume 7 displayed both a high porosity as well as permeability for all segmentation thresholds indicating a high pore connectivity. From these results it can be concluded that both porosity and pore connectivity are heterogeneously distributed across the particle. Nevertheless, most of the particle does not seem to be well connected by macropores. To assess pore connectivity in more detail, the measured pore network was expressed as a pore-network model as previously described by Meirer, Kalirai and co-workers (Fig. S7†).^51^ By doing this, the pore volume is described by a set of points, lines, and corresponding distances to pore boundaries. Fig. 5 displays the pore network model of the MOF assuming total porosities of 2.6% (a), 18% (b) and 29% (c). Here it can clearly be seen how the macropore connectivity increases with increasing porosity and goes along with the appearance of pore channels with larger diameters (red regions Fig. 5b and c).

**Fig. 6:**
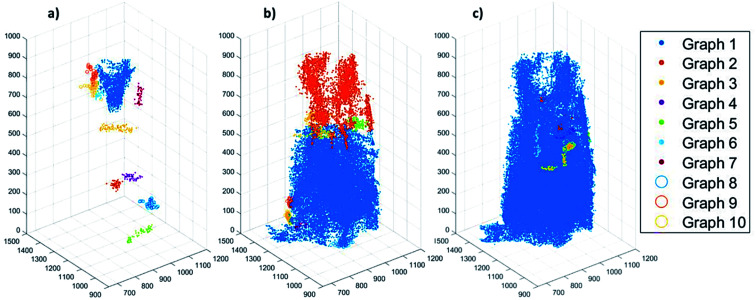
Plots showing the 10 pore-network graphs (subsets of pore networks that are not connected with each other) with the largest volumes for different segmentation thresholds based on the different total porosities of (a) 2.6%, (b) 18% and (c) 29%. The results for the other considered thresholds can be found in Fig. S8 of the ESI.†

To further evaluate connectivity, the different graphs (defined as a group of connected segment points), were analyzed separately using Matlab®. The 10 largest graphs obtained for each intensity threshold are plotted in Fig. 6. By binarizing the image based on Hg-porosimetry (29% porosity), the whole particle volume is well connected by the biggest sub-network (blue dots). At a total porosity of 18%, on the other hand, the MOF pores are not entirely connected by a single network, but the first two graphs combined (orange and blue dots). Note that two graphs are never connected to each other. In these two cases, the macro pore network we map here would have a significant effect on mass transfer, creating diffusion “highways” and strongly reducing the separation properties of the MOF. However, for a total porosity of 2.6% (and also for the case 5% porosity, see Fig. S8†) all sub networks are very much localized, that is, macro-pore connectivity is low, which is desired for separation processes. The porosity value of 2.6% determined above as the best estimate for this individual crystal indicates that any diffusing medium will necessarily flow through a certain fraction of micropores, which is of the upmost importance since MOFs usually rely on the functionalities present therein (*e.g.* coordinatively unsaturated Lewis sites, OH groups or on-purpose attached organic moieties) for molecular sorting.^9,36,37^ Hence, the findings presented up to here have two implications: they indicate that MIL-47(V) crystals synthesized by hydrothermal methods contain macroporous void regions (2–3% of the crystal volume) as crystallization defects. However, the connection between these macro-porous regions is not sufficient to create diffusion pathways that result in a significant loss of the MOFs sieving properties.

### Graph orientation

**Fig. 7:**
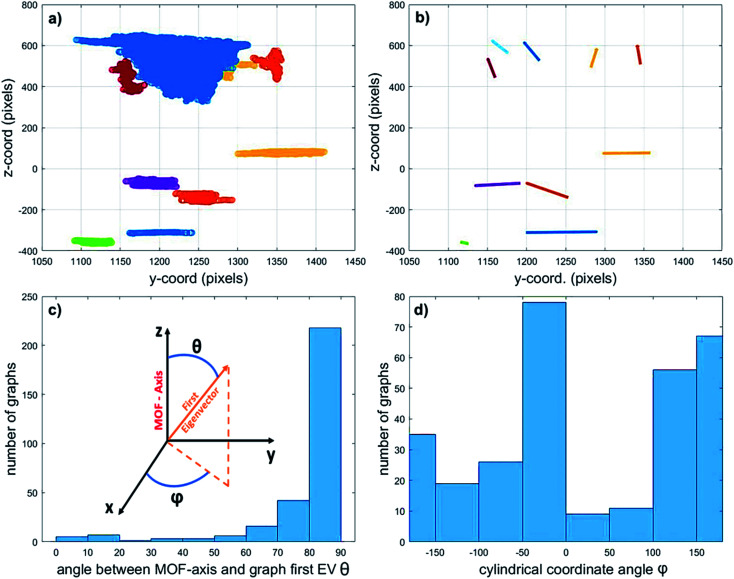
(a) The 10 sub-networks with the largest volumes assuming 2.6% total porosity of the individual MOF crystal. (b) Corresponding first eigenvectors (EVs) normalized and projected to the *z*–*y* plane (the apparent length difference is caused by the projection onto the *z*–*y* plane). The position of each EV corresponds to the average coordinates of each data cloud (graph) it refers to. (c) Histogram of angles between each graph's first eigenvector and the main MOF axis (*θ*). Spherical coordinates of the first EVs of the sub-networks. The 3D volume was rotated, so that the MOF axis is parallel to *z*. *θ* describes how parallel the EV is with respect to the main MOF-axis, *Φ* describes the orientation of its projection onto the *x*–*y* plane. (d) First EV orientation histogram projected onto the *x*–*y* plane (angle *φ*).

Finally, in an attempt to elucidate the origin of the detected macropores, their orientation in relation to the MOF axis was assessed by calculating the first eigenvector (EV) of the covariance matrix of each graph (*i.e.* connected pore sub-network). This vector points in the direction of the greatest spread of each graph (expressed as a cloud of points in space) and can therefore be used as a measure for pore orientation. Fig. 7 exemplifies this method by displaying the 10 sub-networks with the highest volume (a) and their corresponding first EVs (b). The position of the displayed EV corresponds to the average coordinates of the graph (point cloud) they describe.

The inset in Fig. 7c reports the spherical coordinates of each Eigenvector, that is, the angles between each vector and the main MOF axis (as defined above, *z*-direction in the plot). These angles describe how parallel the graph orientation is with respect to the MOF axis (angle *θ*) and how the sub-network is oriented in the *x*–*y* plane (angle *φ*). Fig. 7c and d display the orientation of the first EV of the biggest 300 sub-networks assuming a total porosity of 2.6%. The histograms report the *θ* (c) and *φ* (d) angles between the first EV of the graphs and the MOF axis. Based on these results there does not seem to be a clear preferred pore network directionality when inspecting the EVs that are projected perpendicularly to the MOF axis (Fig. 7d). On the other hand, the left histogram (Fig. 7c) shows that most of the graphs have an angle *θ* between 80° and 90°. This means that most of the macropores are oriented perpendicular to the main MOF axis. Such preferential defect directionality suggests that the main crystal growth takes place along this direction. As the crystal grows, small defects can be amplified to become radially oriented macropores like the ones observed here. Similar results were obtained for all other total porosity values studied (see ESI Fig. S9†). However, as the total porosity grows, there is a slight shift towards smaller angles in the *θ*-angle histogram. This is due to an increase in connectivity along the MOF axis when the assumed porosity increases (see Fig. S8†). The growth of such aligned defect domains is common in MOF crystals^59–61^ and is a potential explanation for the presence and orientation of the voids observed. Further *in situ* imaging studies of a growing crystal would be necessary to confirm this hypothesis.

## Conclusions

This study shows that it is possible to map the macropore defects of a single MIL-47(V) MOF crystal with X-ray nanotomography. This method represents a non-destructive way of studying the internal pore space of MOF crystals and to characterize their internal architecture. In contrast to Hg-intrusion, TXM allowed to discriminate between porosity corresponding to internal voids, around 2–3% of the volume, and the interparticle space. The intrusion experiments clearly overestimated macro-porosity, rendering TXM a powerful alternative when detailed information of the internal macro-porosity is needed. Further numerical processing revealed that the observed macropores are not distributed homogeneously across the particle and their connectivity seems to be very poor. Also, as demonstrated by permeability simulations, they presumably only have a limited effect on mass transfer and therefore on the separation properties of the crystal for mixtures flowing through the MOF's internal pore space. By analysing the tilting angle of the pore regions, a preferred network orientation was observed. The pore sub-networks are mostly spread perpendicularly to the main MOF-axis, which could lead to anisotropies in diffusion and permeability. As the exact total porosity of the sample can only be estimated by cross-validating results from Hg-porosimetry, FIB-SEM, and X-ray nanotomography, it is not yet possible to quantify the exact extent of these effects.

This is in line with studies from different fields that compare X-ray tomography with conventional pore analysis methods,^55,62–65^ and highlights the added value of using X-ray microscopy as a non-destructive tool for MOF materials. This work is the first example of imaging of the inner macro-pore space of MOF materials at the single particle level and we predict that it will trigger further investigations of the internal porosity of other important topologies and morphologies.

## Experimental

### Synthesis of MIL-47(V)

Hydrothermal preparation of the crystals was carried out as described by Barthelet *et al.*^69^ In short, vanadium metal powder (Sigma-Aldrich, 99.9%), terephthalic acid (Sigma-Aldrich, +98%), hydrofluoric acid (Sigma-Aldrich, 48–51 %wt aq. solution), and DI water (molar ratio 1 : 0.25 : 2 : 250) were introduced in a Teflon-lined Parr steel autoclave for four days in an isothermal oven at 473 K (autogenous pressure, filling rate: 50%). The green-yellow solid was collected by centrifugation and washed 3 times with DMF and ethanol (50 mL), then dried at 423 K under vacuum for 24 h. Powder X-Ray Diffraction (PXRD) (Fig. S10†) was performed using a Bruker-AXS D2 Phaser powder X-ray diffractometer in Bragg–Brentano geometry, using Co K_α1,2_ = 1.79026 Å, operated at 30 kV. Measurements were carried out between 5 and 70° using a step size of 0.05° and a scan speed of 1 s.

### Hg-porosimetry

MIL-47(V) crystals (*m* = 0.634 g) were outgassed for 24 h in air flow at 150 °C prior to loading them in the tube. Intrusion experiments were performed using an Hg-porosimeter Micromeritics Autopore IV 9510 in the pressure range *p* = 0.05 to 420 MPa. We used Washburn's equation (*p* = −4*γ* cos *θ* × *d*^−1^), with *γ*, mercury surface tension, and *θ*, contact angle, with values of 0.485 N m^−1^ and 130°, respectively, to calculate the pore diameter, *d*.

### Focused ion beam-scanning electron microscopy

Prior to measurements, the sample was coated with a Pt/Pd layer (∼10 nm) with a Cressington HQ280 sputter coater. Measurements were performed with a FEI Helios Nanolab 600 FIB-SEM instrument. The sample was placed on an aluminum stub using a carbon sticker. A protective layer of Pt (∼3 μm) was deposited on top of the region of interest before performing the measurements. For the FIB experiments, a trench was made by milling perpendicularly to the surface, next to the Pt-deposited area. After milling the trench, a cleaning step with Ga^+^ ions were performed before imaging. SEM images of the cross section were recorded in backscatter electron (BSE) mode (2 kV, 50 pA) and in secondary electron mode (2 kV, 0.1 nA).

### Transmission X-ray microscopy tomography data collection and 3D reconstruction

Full-field transmission hard X-ray microscopy (TXM) was performed at beamline 6-2C of the Stanford Synchrotron Radiation Lightsource at the Synchrotron Linear Acceleration Center (SLAC) National Accelerator Laboratory. Details of the experimental setup can be found elsewhere.^66,67^ The beam energy was calibrated by measuring at the V K-edge of a reference metal foil. X-ray nano tomography was conducted below and above the V K-edge energies (5460.0 and 5482.0 eV) five times per angle with an angular step size of 1 degree over a range of 180 degrees, enabling a high-quality reconstruction of the 3D structure of the MOF single-crystal with the TXM-Wizard software package.^59^ The total duration of the scan was 260 minutes. Data analysis, processing and sample preparation details are further described in the ESI† and in the Results and discussion sections.

### Transmission X-ray microscopy data analysis

Each stack of 2-D projection images was aligned manually to correct for motor jitter and sample movement. Later, the 3-D tomographic slices were reconstructed with an iterative algebraic reconstruction technique (iART). TXM tomography data was binned from a 32 nm isotropic voxel size to a voxel size of 64 × 64 × 64 nm^3^. The effective 3D spatial resolution was estimated to 230 nm by Fourier Shell Correlation (FSC) analysis of the tomography data (see ESI, Fig. S11†),^68^ therefore micro- (<2 nm), mesopores (2–50 nm) and small macropores could not be resolved.^39^ Each slice of the obtained 3D image was segmented manually, to determine the total particle volume (including pores) using Avizo Fire© software. Further details can be found in the Results and discussion and the ESI sections.†

## Author contributions

R. M. G. (Utrecht University, UU) reconstructed and analyzed the TXM tomography in discussion with F. M. (UU) and R. V. (UU); R. M. G. (UU) together with M. R. T. (UU) drafted the manuscript. M. R. T. and K. W. B. (UU) prepared the materials and collected XRD, TXM and routine data. N. N. (UU) carried out the FIB-SEM imaging of the cross-sections of the large crystals. K. W. B., B. S. (UU), F. M. and B. M. W. contributed to the proposal for beamtime and K. W. B., J. N.-W. (SSRL) and B. S. contributed to collecting the data at SSRL beamline 6-2c. J. Y. (ICV-CSIC) provided valuable input and performed the Hg-porosimetry measurements. F. M. and B. M. W. (UU) supervised the research and helped in the preparation of the article.

## Conflicts of interest

The authors declare no competing or financial interests.

## Supplementary Material

SC-012-D1SC00607J-s001
