# Supplementary material for: Visualizing defects and pore connectivity within metal–organic frameworks by X-ray transmission tomography
Source: Chem Sci. 2021 May 6;12(24):8458–67. doi: 10.1039/d1sc00607j (PMC8221180; doi:10.1039/d1sc00607j)
Supplement: SC-012-D1SC00607J-s001 [file SC-012-D1SC00607J-s001.pdf]

Electronic Supplementary Material (ESI) for Chemical Science.
This journal is © The Royal Society of Chemistry 2021

SUPPLEMENTARY INFORMATION

Visualizing Defects and Connectivity within Metal Organic Frameworks by X-ray Transmission Tomography

Rafael Mayorga- González[a‡], Miguel Rivera-Torrente[a‡], Nikolaos Nikolopoulos[a], Koen W. Bossers[a], Roozbeh Valadian[a], Joaquín Yus[b], Beatriz Seoane[a], Bert M. Weckhuysen*[a], Florian Meirer*[a]

[a]Inorganic Chemistry and Catalysis Group, Debye Institute for Nanomaterials Science, Utrecht University, Universiteitsweg 99, 3584 CG Utrecht, The Netherlands
[b]Instituto de Cerámica y Vidrio, Consejo Superior de Investigaciones Científicas (CSIC), Kelsen 5, 28049 Madrid, Spain

‡ These authors contributed equally to this work.

Corresponding author: f.meirer@uu.nl, b.m.weckhuysen@uu.nl

Table of Contents

1. **Hg Intrusion Experiments for Porosimetry**
2. **Focused Ion Beam Scanning Electron Microscopy**
3. **Range of Intensity Thresholds**
4. **Permeability Simulations**
5. **Numerical Solid Expansion for Analysis of Volume Porosity**
6. **Pore Size Distribution**
7. **Connectivity and Thresholding**
8. **Geometry Parameters of 3D Reconstructed MOF Crystal**
9. **Graph Orientation**
10. **X-ray Diffraction of MIL-47(V)**
11. **Fourier Shell Correlation Analysis**
12. **High Intensity Regions**
13. **References**
14. **qualitative sub-resolution crystal defect assessment**
15. **TXM sample preparation**

1. Hg Intrusion Experiments for Porosimetry

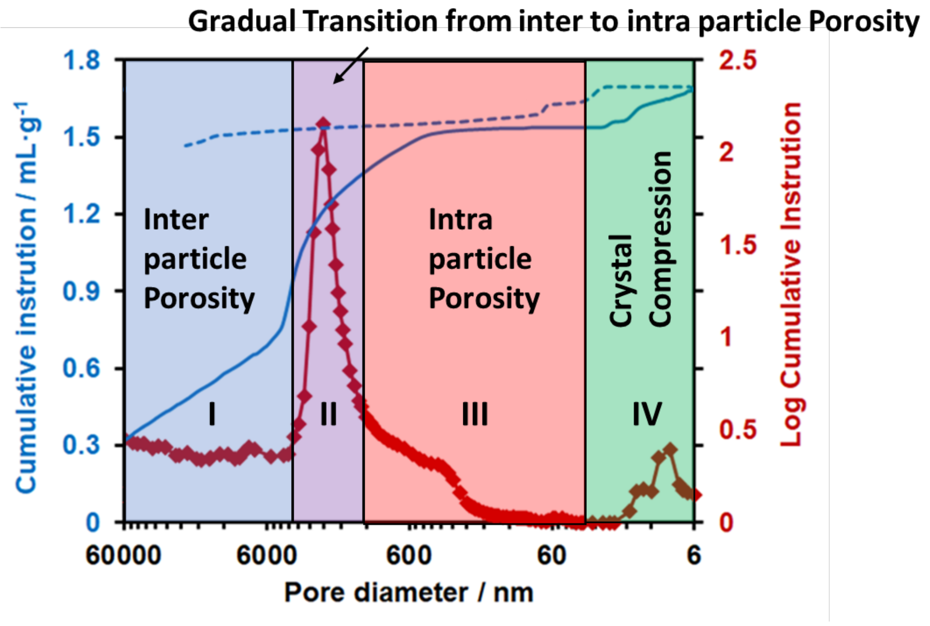

Figure S1. Cumulative and incremental volumes of intruded mercury as a function of the effective pore diameter. The steep increase in Hg cumulative intrusion corresponding to pores in the 6×10^3 to 6×10^4 nm range is associated to interparticle porosity (region I). Here, voids within and in between particles are filled with mercury. The greater the intrusion pores of this regime, the more inter particle porosity is being probed (region II). Conversely, when smaller pores are filled, they are more likely to be a consequence of intra particle porosity. The rather small mesopores measured in region IV are a result of a compression of the crystal due to high pressures.[1] The described regimes cannot be quantified during this experiment, this figure displays their ranges only qualitatively.

In Figure S1. Cumulative and incremental volumes of intruded mercury as a function of the effectivecumulative and incremental volumes of intruded mercury as a function of the effective, mercury cumulative intrusion and pore size distribution obtained thereof, shows the presence of two peaks at around 0.1 (~2-3·10^3 nm, region II in Figure S1) and 100 MPa (~8-6 nm, region IV Figure S1). The latter has been previously assigned to the reversible compression of the lattice, due to the poor wetting of the MOF surface, preventing Hg from penetrating the micropores and the former, has been ascribed to space between crystals.[1] The minimum pore diameter which may be probed at 420 MPa is 2.96 nm, *as per* Washburn's equation (γ = 0.485 mN·m^{-1}, θ = 130º).

However, as seen in Figure 3a of the Main Text, cross-sections of different parts of the crystal show areas of lower X-ray absorption intensity, exhibiting macropores that are up to 2-3 µm in size. This is of great importance, because both bulk and intraparticle pores with such dimensions could be present in region II of Figure S1. Porosity was determined as the percentage of volume that can be intruded with Hg between 0.2 and 20 MPa, which represents the adsorption plateau at which the larger pores and interparticle space have been filled with Hg but before mechanical compression:

$$\left(\frac{V_{Hg}(0.2\ MPa)}{V_{Hg}(20\ MPa)}\right) \times 100 = \frac{0.43}{1.51} \times 100 = 28.47\ \%$$

2. Focused Ion Beam Scanning Electron Microscopy

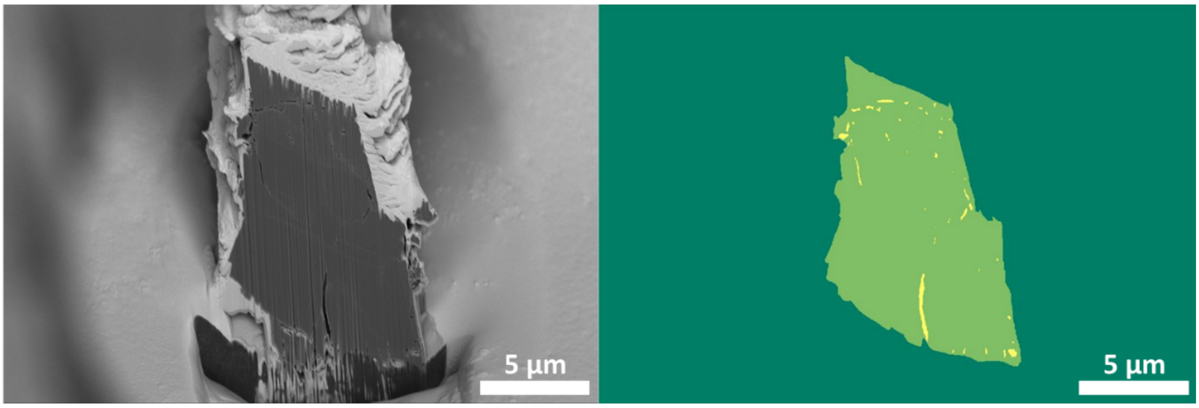

Figure S2. Manual Segmentation of Cross Section and pores. The fraction of yellow pixels (pores) within the light green area (particle cross section) corresponds to the porosity estimated from these data.

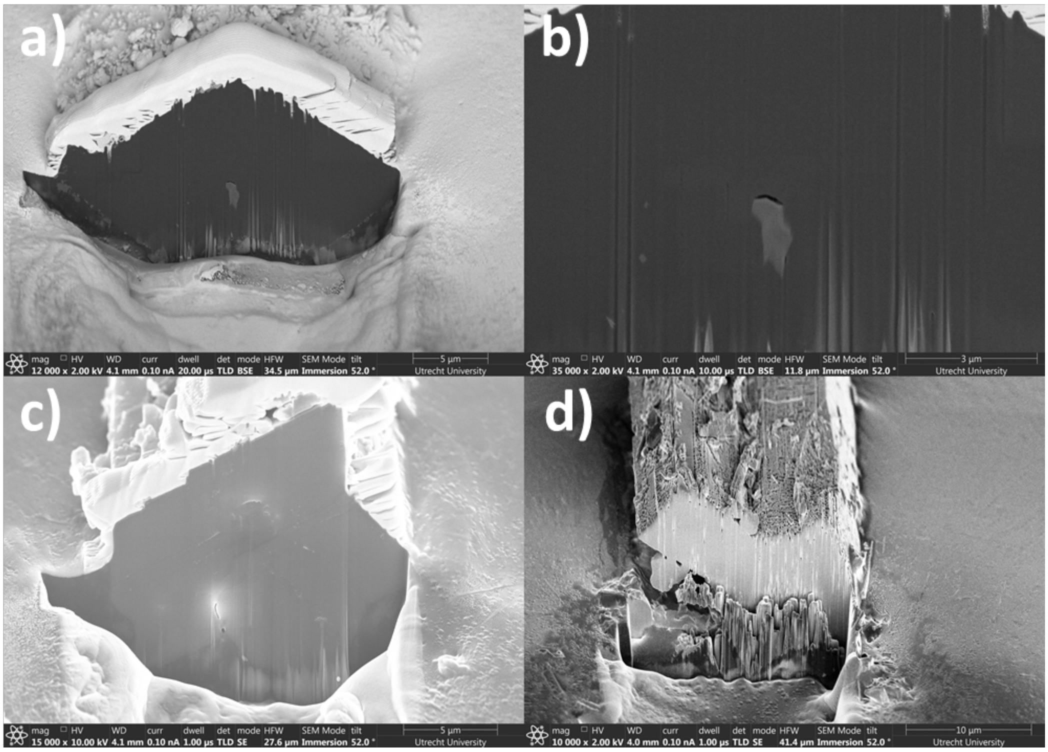

Figure S3. Examples of other cross sections. (a) Cross section with a relatively low porosity. (b) Zoom in of (a) In the center of the crystal, a *denser* area can be observed next to a macropore. (c) Material cross section imaged with secondary electrons accentuating macropores (lighter regions). (d) Crystal cross section displaying few defects.

3. Range of Intensity Thresholds

Since Hg-porosimetry seemed to overestimate the total porosity of the MOF crystal, two other intensity thresholds were evaluated based on the image background intensity histogram. One of them corresponds to the maximum background intensity and resulted in a total porosity of 18 %, while the other was chosen based on the mean value of the background intensity distribution leading to 2.6 % void fraction (Figure S4). Due to the uncertainty in terms of porosity, several grayscale thresholds were considered and compared in this study, that is, between the three values indicating the boundary conditions for threshold values (with corresponding porosities: 29, 25, 20, 18, 15, 10, 5, 2.6 %). Figure S4 depicts the binarized images resulting from all studied thresholds for an arbitrarily selected cross section.

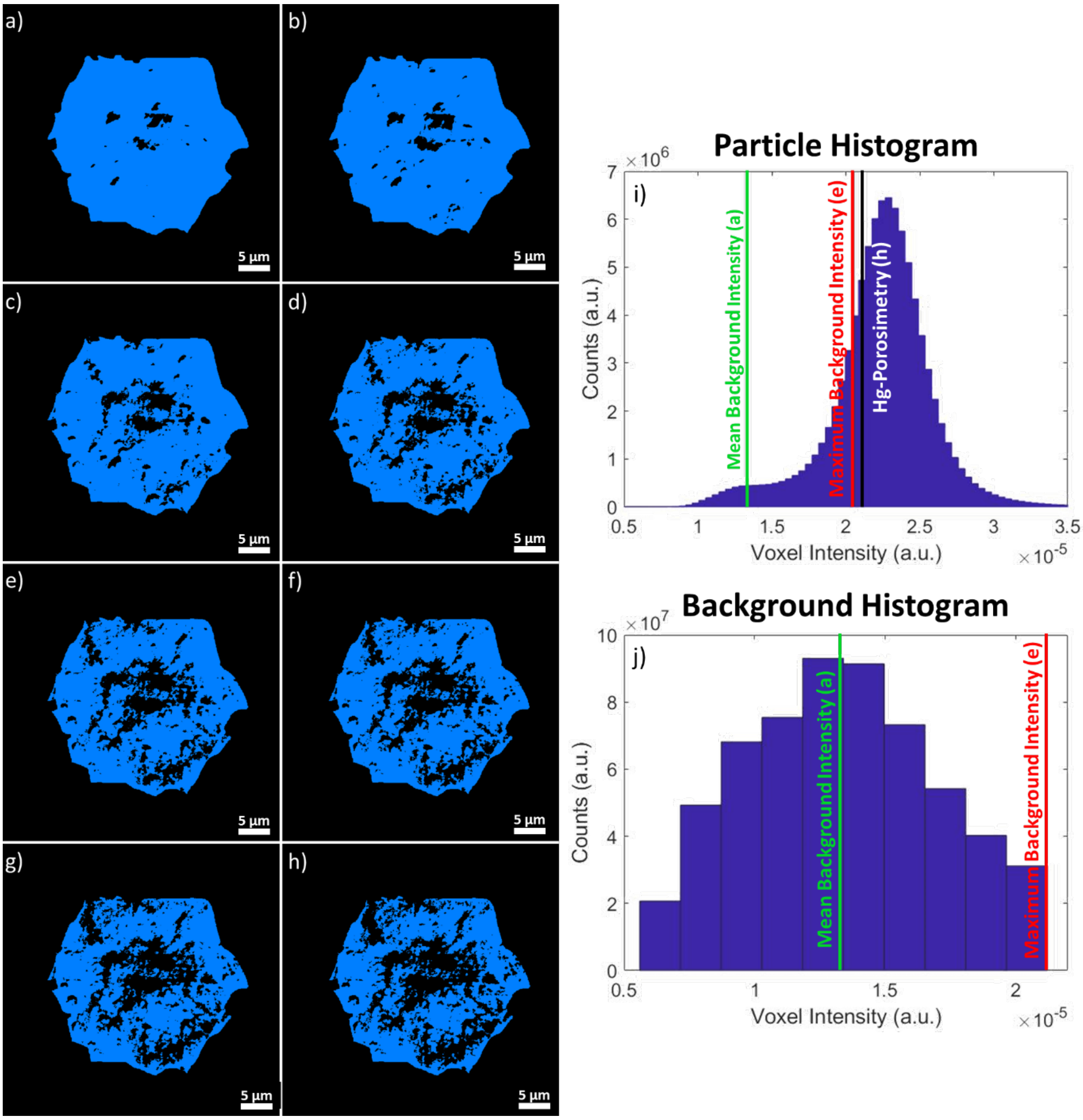

Figure S4. Segmented images of a cross section of the MOF crystal assuming different total porosities. 2.6, 5, 10, 15, 18, 20, 25, 29 % (a-h). (i) Grayscale intensity histogram of the particle displaying thresholds corresponding to 2.6, 18 and 29 % total porosity. (j) Grayscale intensity histogram of the image background displaying the thresholds corresponding to 2.6 %, 18 % total porosity.

In order to determine the porosity of the binarized images the following formula was used for each of them:

$$\text{porosity} = \frac{\text{number of solid voxels}}{\text{number of solid voxels} + \text{number of void voxels}}$$

4. Numerical Solid Expansion for Analysis of Volume Porosity

A pore throat analysis as described by Yang *et al.*[2] was performed: the solid phase of a binarized image is expanded layer by layer iteratively. This causes the blockage of pore throats resulting in isolated pores. After each iteration step, the number of closed pore throats is counted. This number is related to the amount of pore bottlenecks with a diameter as great as the current solid expansion thickness. Therefore, it can be used to study the pore throat size distribution. This procedure is illustrated schematically in **Error! Reference source not found.**S5.

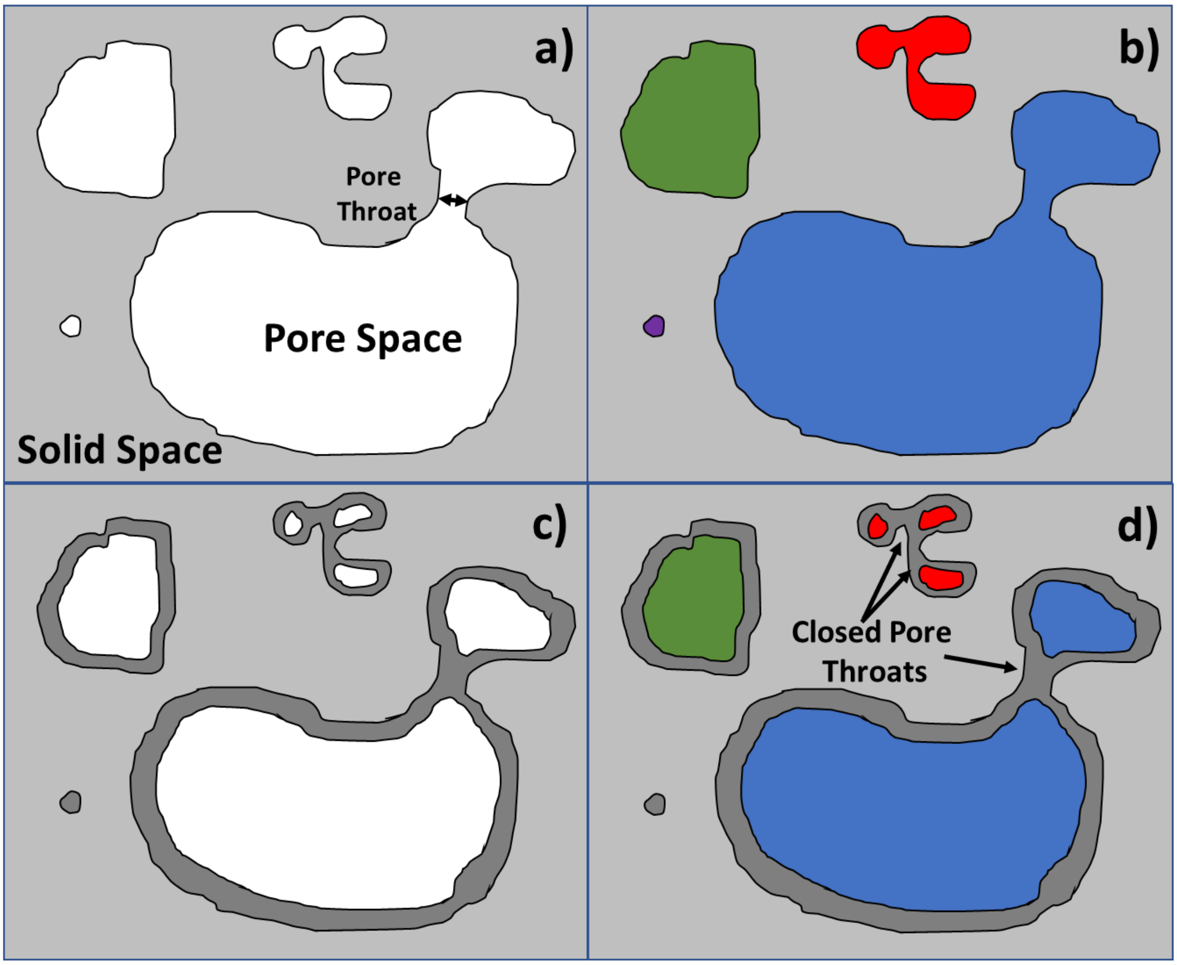

Figure S5. Schematic illustration of the numerical solid expansion algorithm. Step 1: Pore space (a) is labelled leading to (b). Step 2: layer by layer solid space expansion (c) Step 3: Masking of (b) with (c) leads to image (d). Step 4: Counting of closed pore throats; Purple: 1; Blue: 1; red: 3; green: total: 5.

5. Pore Size Distribution

The pore (throat) size distribution was evaluated experimentally with Hg-porosimetry and computationally by numerical solid expansion (Figure S) as well as pore network modelling (Figure S7). The chosen intensity threshold was based on the Hg-porosimetry data (29% porosity). Nevertheless, the computational methods show a peak in the size distribution below 200 nm. According to Hg-porosimetry, on the other hand, this value should be around 1 µm (see Figure S1). Although some pores in this size range can be observed with TXM tomography, most of them are smaller.

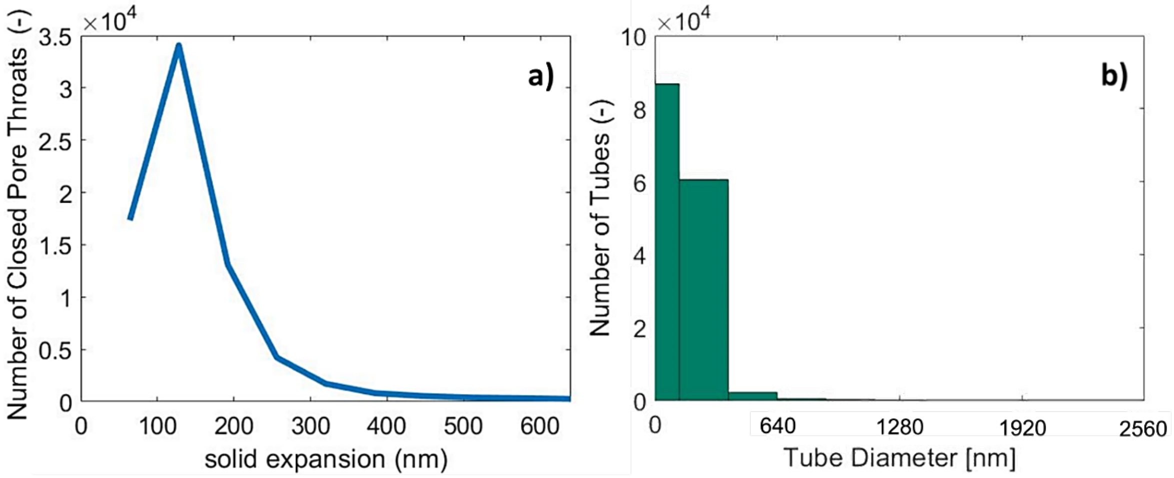

Figure S6. Estimations of Pore size distribution with different methods assuming a value of 29 % total porosity of the analysed volume: (a) numerical solid expansion (b) model cylinder diameter distribution. Note that there are pores smaller than the voxel size, however that evaluation is out of the scope of this study. The peaks of both pore size distributions are much lower than the one obtained with Hg-porosimetry (*ca.* 1 µm).

6. Permeability Simulations

Permeability simulations in (see Figure 4b in the main text) were carried out on each sub-volume using Avizo® XLabHydro. This software estimates the steady state velocity field of an incompressible fluid (water) by numerically solving the continuity and Navier-Stokes equations:

$$\nabla \cdot \vec{u} = 0$$

$$\mu \nabla^2 \vec{u} - \Delta p = 0$$

where μ is the medium viscosity, u its velocity and Δp is the pressure gradient of the causing the flow. The permeability is determined using Darcy's Law:

$$Q = \frac{kA\Delta p}{\mu L}$$

where Q is the global volume flow rate of the fluid, k is permeability and A and L are the cross-sectional area and the length of the sample volume respectively.

7. Pore Network Modelling

The void space was represented by its topological skeleton i.e. a thinned version of the pore shape. After skeletonization, the pore volume is described by a set of points, lines, and corresponding distances to pore boundaries. The skeletonization of the segmented pore volumes was performed using the Avizo© XSkeleton Pack software. The distance of every pore voxel to its closest boundary was calculated and then voxels were removed one by one from the segmented object until only a string

of connected voxels remained. These resulting lines were then translated into points, segments (connecting points), and nodes (points where more than two lines meet) forming a topological model of the pore-network (see Figure S7). Each pore channel can be represented by a cylinder, in which the height is the distance between two segment points and the radius is their average distance to the solid walls. By summing the volumes of these cylinders, the total volume of the pore network can be computed.

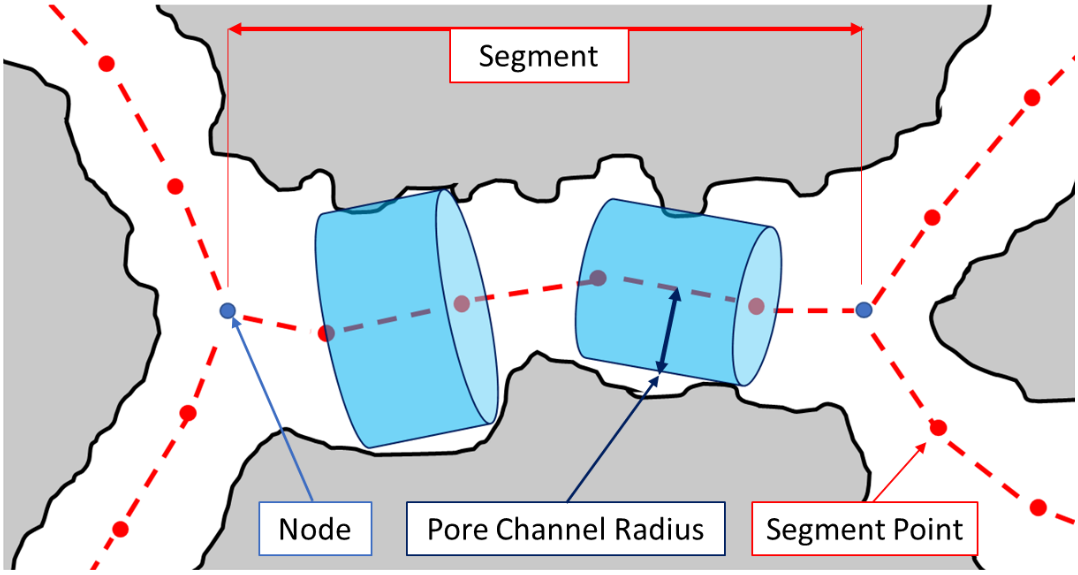

Figure S7. Schematics of the topological representation of the pore network. The measured pore space is modelled by a set of segment points, segments, nodes, and pore channel radii.

8. Connectivity and Thresholding

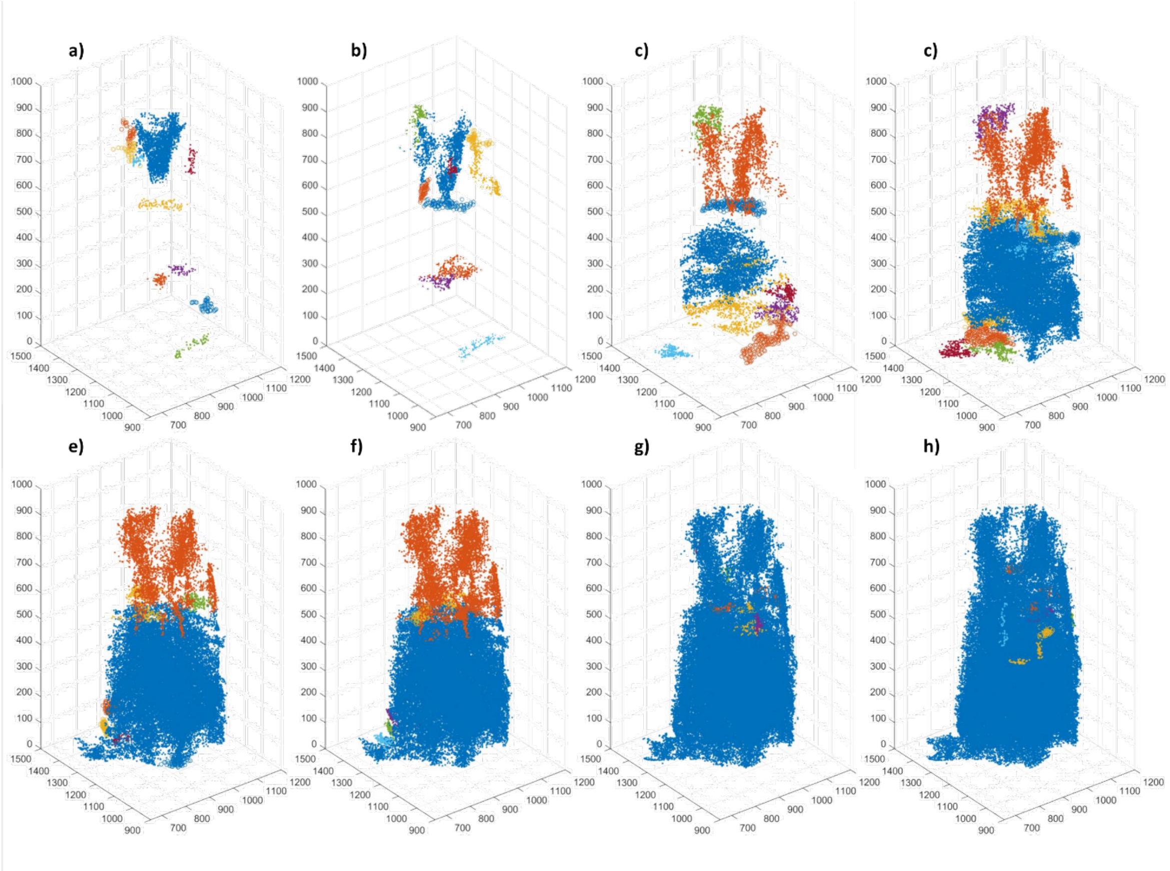

Figure S8. Plots with 10 graphs with the greatest volumes for different total porosities: 2.6 %, 5 %, 10 %, 15 %, 18 % ,20 %, 25 % and 29 % (a-h). The higher the total porosity the higher the connectivity of the macropore sub-networks. At total porosities between 2.6 % and 5%, the subnetworks are very localized and poorly connected.

Based on the image histogram of the absorption intensity background of the transmission X-ray tomography (Figure S4), as well as visual inspection of the segmented cross sections (Figure 3) and the porosity estimated from the FIB-SEM (< 2 %), we suggest that the total porosity value should be closer to 2.6 %. This would mean that the macropore defects have theoretically little effect on altering the transport properties predicted to happen through the micropores of the MOF-crystal (even more so given the poor connectivity of these pores). However, it is worth mentioning that this seemingly isolated macropore networks could be connected by smaller macropores that could not be resolved with the TXM-tomography. Since the measured X-ray absorption is correlated with material density, voxels containing pore defects smaller than the resolution (230.7 nm), led to lower voxel grayscale values compared to their defect-free counterparts. Therefore, when the hypothetical porosity is increased, more voxels containing unresolved pore defects are interpreted as void space and since these pores are not necessarily connected, this leads to an overestimation of connectivity. If the assumed total porosity is gradually increased from 2.6 % to 5 %, the extension of the resulting subnetworks increases (Figure S8), however they are still very localized and unconnected. This suggests that the observed macropore networks are not interconnected by macropore defects that could not be resolved with the TXM-tomography.

9. Geometry Parameters of 3D Reconstructed MOF Crystal

Table S1. Different geometrical parameters of the solid volume comprising the MOF crystal.

**Total Particle Volume (μm³)**	26366.1
**Anisotropy (-)**	0.907861
**Flatness (-)**	0.906615
**Elongation (-)**	0.101629

$$\text{Anisotropy} = 1 - \frac{\text{Minimum Eigenvalue of datacloud}}{\text{Maximum Eigenvalue of datacloud}}$$

$$\text{Flatness} = \frac{\text{Minimum Eigenvalue of datacloud}}{\text{Medium Eigenvalue of datacloud}}$$

$$\text{Elongation} = \frac{\text{Medium Eigenvalue of datacloud}}{\text{Maximum Eigenvalue of datacloud}}$$

10.Graph Orientation

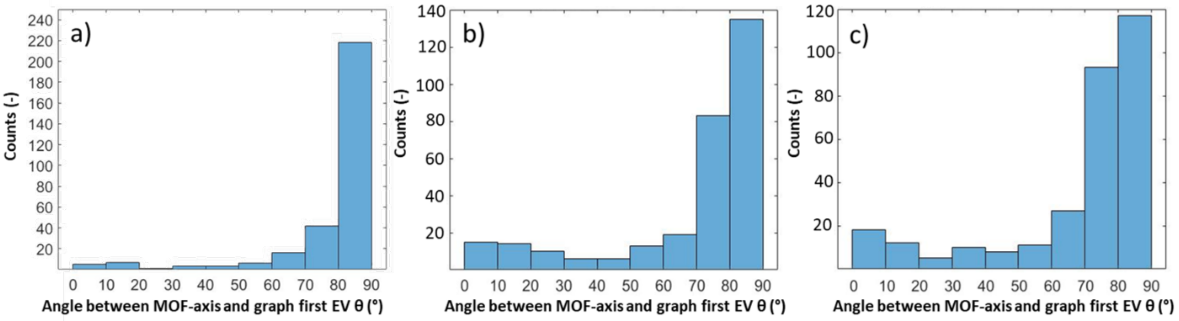

Figure S9. Histogram of angles between Graph first eigenvector and the MOF axis (θ) assuming a total porosity of (a) 2.6, (b) 18 and (c) 29 %. As the porosity increases, there is a slight shift towards the left in the θ-angle histogram due to an increase in connectivity along the MOF axis.

11. X-ray Diffraction of MIL-47(V)

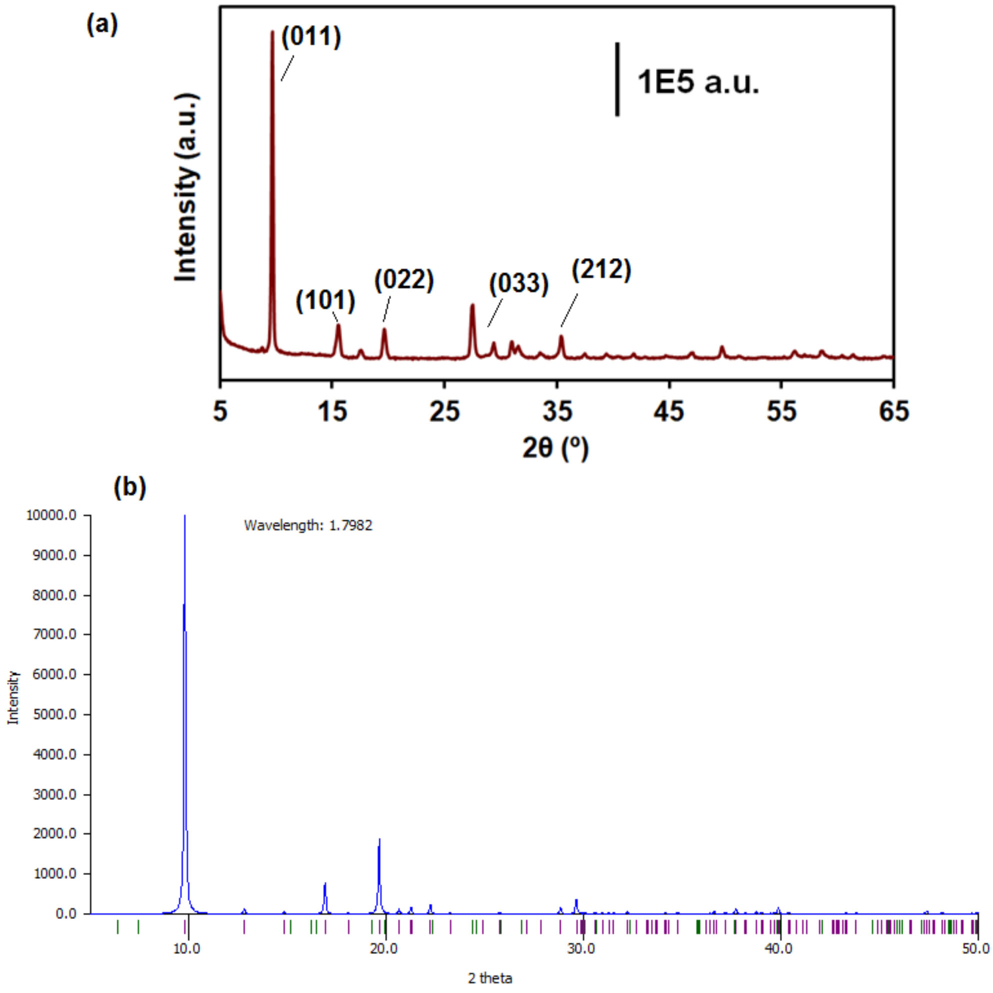

Figure S10. (a) Experimental X-ray diffraction (XRD) pattern of MIL-47(V) as synthesized powder; and (b) simulated pattern from Mercury 3.7 (FWMH = 0.1, K_{α} = 1.7892) with the *.cif* file reported in reference [3].

12. Fourier Shell Correlation (FSC) Analysis

A Fourier Shell correlation (FRC) was performed to estimate the 3D resolution. Therefore, the projection images of the tomography data were separated into two different datasets. One of the datasets contains all images recorded at even angles and the other corresponds to odd recording angles. The analysis was performed for 400 slices of the reconstructed odd and even datasets as described by van Heel and Schatz.[4] Fig. S11 displays the average FSC of all 400 slice pairs. The resolution was determined using the intersection point of the FRC curve with the ½ bit curve. Using this criterion, the resolution calculated was 230.7 nm (0.277 reciprocal pixels of 32 nm size).

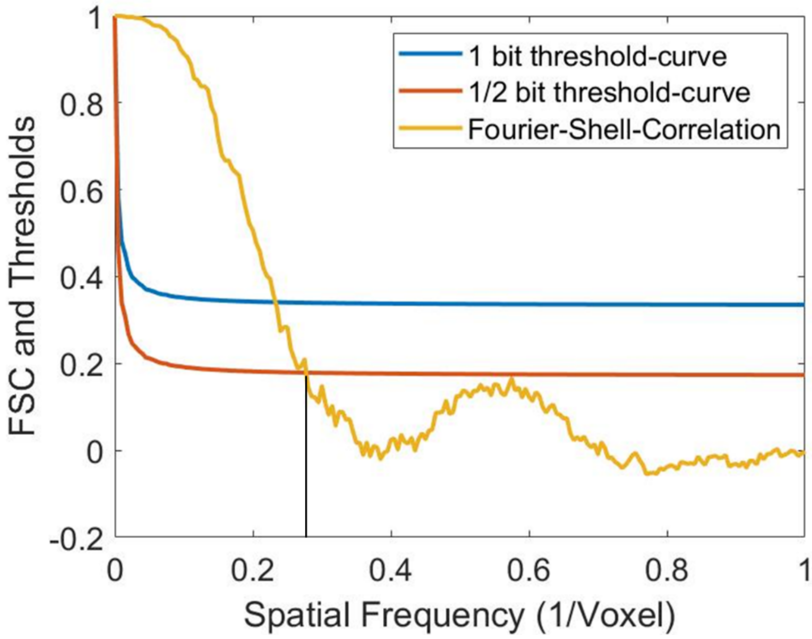

Figure S11. Fourier Shell Correlation (FSC) analysis of the tomography 400 arbitrarily selected slices of the reconstructed data.[4] The intersection at 0.277 reciprocal pixels corresponds to an estimated 3D spatial resolution was 230.7 nm (1/2 bit).

13. High Intensity Regions

Several intensity outlying regions, such as the one displayed in Figure S12 a) were found during our analysis. In order to evaluate those regions, an intensity threshold that surpassed most of the voxels was selected for binarization (see in Figure S12 b, c). The volume fraction of these clusters was about 0.1%, and Figure S12 d) shows the amount of high intensity regions at different distances from the surface. Most of these regions can be found at the surface of the MOF and are possibly explained by defect formation during crystal growth. The farther away from the crystallization nucleus, the greater the probability of finding crystal defects, such as uncoordinated metal clusters or pores. To maintain the material balance in these areas, nanosized metal or metal oxide, *e.g.* metallic V or V_2O_5, clusters are likely to be formed, leading to these changes in X-ray absorption intensity. Similar findings were recently reported by Ferreira-Sanchez *et al.* on HKUST-1 MOFs.[5]

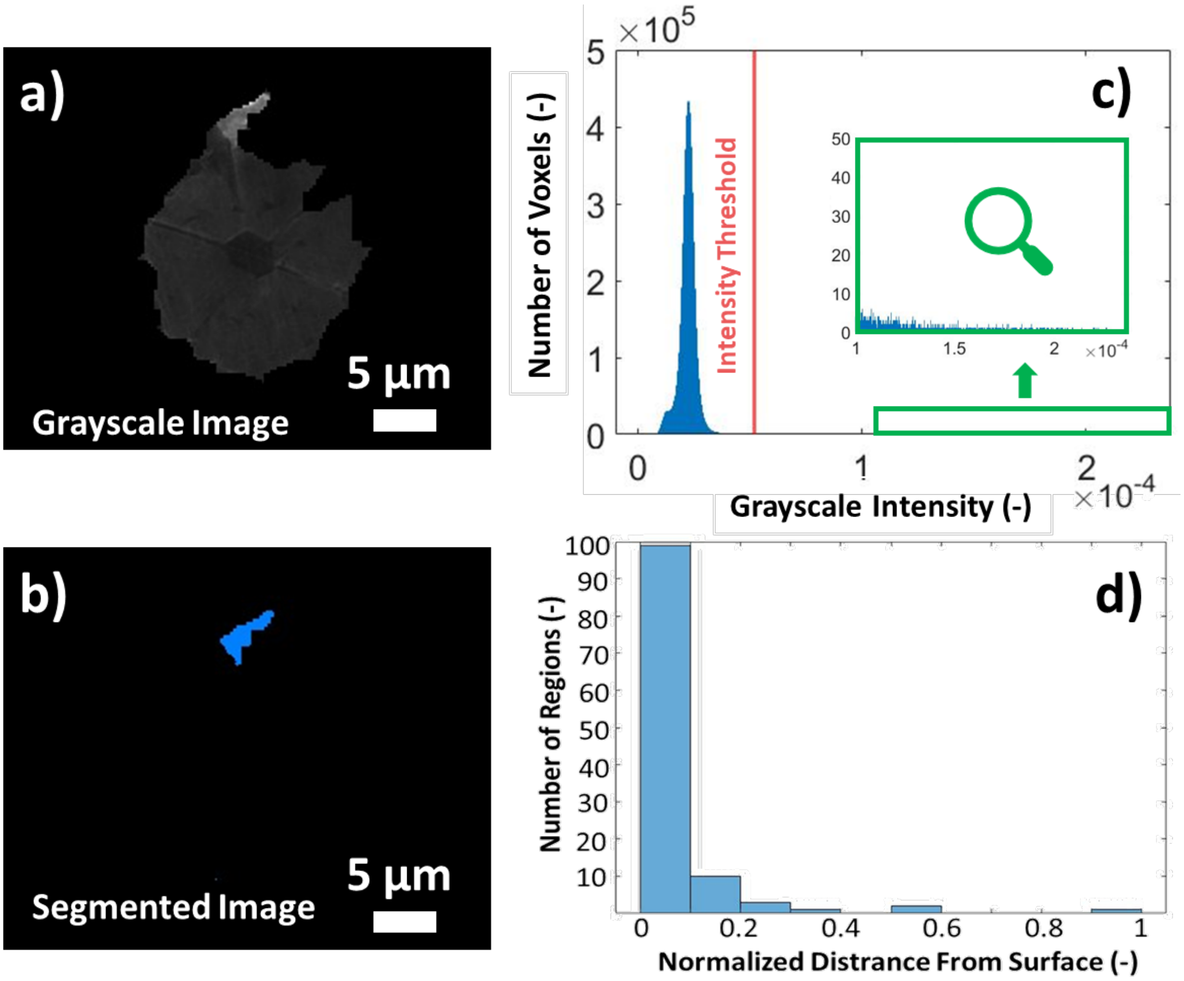

Figure S12. (a) Grayscale image displaying an outlier intensity region. (b) Corresponding binarized cross section. (c) Grayscale intensity histogram of the MOF tomography displaying the chosen binarization threshold. Zoom in at intensities higher than the threshold shows the presence of high intensity voxels. (d) Number of intensity outlying regions as a function of distance from surface. The position of each region corresponds to its centre of mass.

14. qualitative sub-resolution crystal defect assessment

The plot shown in Figure 1 e) shows the normalized mean intensity as a function of the distance to the surface of the particle. These values were calculated in the following way. first, the total pore volume (volume of particle + apparent pores) of the particle was segmented manually from the 3D raw image. Then, the intensities of the surface voxels (and their mean value) were calculated. Next, the surface voxels were removed layer by layer and after each removal step the intensities of the surface voxels (and their mean value) were calculated. Figure S 13 shows a scatter plot containing the intensity of each voxel and its corresponding distance from the surface.

The intensities and distances from the surface displayed in Figure 1 e) were min-max scaled so that their values vary from 0 to 1. It is worth mentioning that there are some outlying intensity voxels (see Figure S12) where the intensity is much higher than in the rest of the particle. Therefore, the standard deviation in Figure 1 e) seems negligible compared to the maximum value. However, by comparing the spread in intensities for each distance in Figure S 13, the mentioned decrease in variance towards the center of the particle becomes apparent.

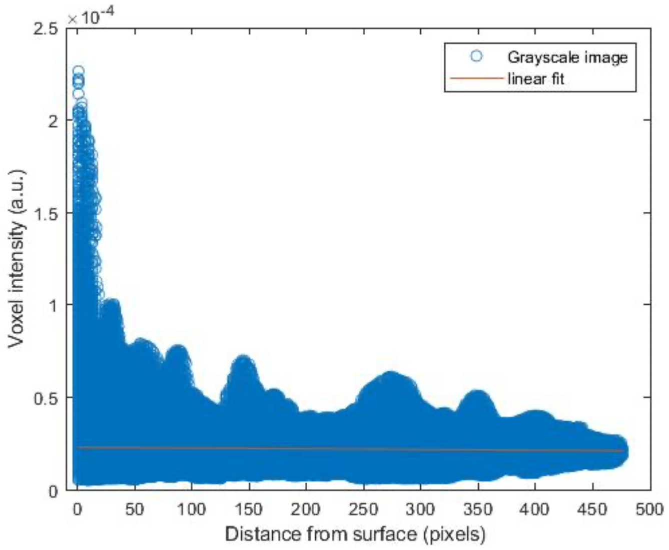

Figure S13. Scatterplot of the intensities of all particle voxels as a function of their distance to the surface. Close to the center of the particle the variance decreases. On the other hand, a linear regression on the datacloud suggests that the mean intensity varies only slightly as a function of the distance from the surface

15. TXM sample preparation

The crystals were suspended in ethanol and loaded into 1 mm diameter Kapton® tubes by capillarity. After loading the samples, the solvent was evaporated and dried in air, after which both ends of the tubes were sealed with epoxy resin. The capillaries were then loaded into glass jars and evacuated in a diaphragm pump connected to a glovebox, where the samples were stored prior to measurement. The tube was loaded onto the rotatory stage at beamline 6-2c, and a He bag with constant 10 mL/min flow at the outlet tube was placed over the sample and X-ray emitting pinholes to prevent air absorbance.

16. References

(1) Yot, P. G.; Ma, Q.; Haines, J.; Yang, Q.; Ghoufi, A.; Devic, T.; Serre, C.; Dmitriev, V.; Férey, G.; Zhong, C.et al. Large breathing of the MOF MIL-47(VIV) under mechanical pressure: a joint experimental–modelling exploration. *Chem. Sci.* **2012,** *3* (4), 1100.

(2) Yang, F.; Hingerl, F. F.; Xiao, X.; Liu, Y.; Wu, Z.; Benson, S. M.; Toney, M. F. Extraction of pore-morphology and capillary pressure curves of porous media from synchrotron-based tomography data. *Sci. Rep.* **2015,** *5* (1), 10635.

(3) Vanpoucke, D. E. P. Linker Functionalization in MIL-47(V)-R Metal–Organic Frameworks: Understanding the Electronic Structure. *J. Phys. Chem. C* **2017,** *121* (14), 8014.

(4) van Heel, M.; Schatz, M. Fourier shell correlation threshold criteria. *J. Struct. Bio.* **2005,** *151* (3), 250.

(5) Ferreira Sanchez, D.; Ihli, J.; Zhang, D.; Rohrbach, T.; Zimmermann, P.; Lee, J.; Borca, C. N.; Böhlen, N.; Grolimund, D.; van Bokhoven, J. A.et al. Spatio-Chemical Heterogeneity of Defect-Engineered Metal–Organic Framework Crystals Revealed by Full-Field Tomographic X-ray Absorption Spectroscopy. *Angew. Chem. Int. Ed.* **2021,** https://doi.org/10.1002/anie.202013422.
